# Immune checkpoint Inhibitor related myocarditis reported through the FDA adverse event reporting system: pharmacovigilance trends in reporting and outcomes

**DOI:** 10.3389/fonc.2025.1498817

**Published:** 2025-02-27

**Authors:** David J. Reeves, Kevin Leffers, Vijay U. Rao

**Affiliations:** ^1^ College of Pharmacy and Health Sciences, Butler University, Indianapolis, IN, United States; ^2^ Department of Pharmacy, Franciscan Health Indianapolis, Indianapolis, IN, United States; ^3^ Franciscan Physician Network, Franciscan Health Indianapolis, Indianapolis, IN, United States; ^4^ International CardioOncology Society Center of Excellence, Indiana Heart Physicians, Indianapolis, IN, United States

**Keywords:** immunotherapy, myocarditis, immune checkpoint inhibitors, adverse effects, pharmacovigilance

## Abstract

**Introduction:**

As the use of immune checkpoint inhibitors (ICIs) continues to expand, it is important to be mindful of rare but serious side effects such as myocarditis. Multiple analyses of adverse effect databases have demonstrated an association between ICIs and myocarditis; however, given the rapid implementation of therapeutic use, introduction of multiple new ICIs, and expanding indications, it is unclear if trends are evolving in reporting and outcomes.

**Methods:**

We analyzed the FDA Adverse Event Reporting System to investigate the association between ICIs and myocarditis and trends in myocarditis outcomes among reports submitted between 2012 and the first quarter of 2023.

**Results:**

After removal of duplicate cases, 1,326 myocarditis cases were reported to the database in patients receiving ICIs. Of these, the majority of reported cases were in males (62%) and the median age was 69 years. Consistent with the increase in utilization, the number of cases reported per year increased with each passing year. The reporting odds ratio (ROR) for all ICI drugs included in the analysis was 30.1 (95% confidence interval: 28.4-32.0). RORs for the individual drugs ranged from 12.3 for durvalumab to 168.5 for nivolumab/ relatlimab. The overall fatality rate of all cases was 37%. A significant difference in fatality rate among reported cases was present when comparing outcomes in 2018 and 2022 (45% vs 33%, respectively, p=0.017).

**Discussion:**

Myocarditis continues to be associated with immune checkpoint inhibitors, with the number of reported cases increasing consistent with increasing utilization; however, the outcomes may be improving with less cases being reported as fatal.

## Introduction

Immune checkpoint inhibitors (ICIs) represent a significant advance in the treatment of multiple malignancies. Approved by the United States Food and Drug Administration (FDA) in 2011 for the treatment of unresectable or metastatic melanoma, ipilimumab was the first drug marketed in this class (1). Over the next decade, ten additional agents or combinations (dual ICI therapy) were approved with upwards of 100 indications, including use in the first-line treatment of multiple malignancies, such as non-small cell lung cancer and urothelial carcinoma. Use has even migrated from the treatment of metastatic malignancies to early-stage disease and may be given as monotherapy, in combination with chemotherapy, and more recently, in combination with radiation. Given the significant growth in utilization, there is much interest in the safety of these agents. These drugs exert their anti-cancer effects by suppressing inhibitory signaling mechanisms between tumors and the immune system, particularly T-cells (2). This allows for an immune-mediated destruction of tumors; however, it may also result in immune related destruction of non-malignant (i.e., normal) tissue. Immune related adverse events (iRAE), an extension of ICI therapeutic effects, are the most common side effect and can impact any organ system, including the cardiovascular system. Myocarditis has been described to occur in 0.09-2.4% of patients receiving ICIs and onset is generally within 30 days of treatment initiation (2–4). Risk factors for myocarditis include use of dual ICI therapy and the presence of a concurrent iRAE, where approximately a third of patients have been reported to exhibit concurrent myositis (2, 4).

Due to the relatively rare occurrence of ICI myocarditis as well as difficulty in making the clinical diagnosis, identification in prospective studies has been challenging. Therefore, analyses of adverse event reporting systems have been completed to determine if a reporting signal for myocarditis was present for ICI therapies (5–7). All analyses to date have identified a significant reporting signal for myocarditis with ICI therapies (5–7). In addition, an analysis of reports to the FDA Adverse Event Reporting System (FAERS) from 2004 to 2018 identified a fatality rate of 51% (5). Since this report in 2018, the number of indications for ICIs has expanded with over 60 indications approved by the FDA since the beginning of 2019. As utilization has increased and practitioners have become acquainted with these therapies and their adverse effects, it is unknown if the outcomes of this serious iRAE have improved since 2018. Moreover, it is unknown if there are differences in ICI myocarditis case characteristics and outcomes based on ICI therapeutic target [programmed cell death protein 1 (PD-1), PD ligand 1 (PD-L1), or cytotoxic T-lymphocyte associated protein 4 (CTLA-4)] or with use of combination therapy. In order answer these questions, considering the advances in ICI therapy over the past 5 years, an analysis of FAERS data was undertaken to describe the myocarditis reports associated with ICI agents and the association between ICI and myocarditis reporting, to assess trends in reported myocarditis outcomes over the years, and assess differences in outcomes based on ICI therapeutic target.

## Materials and methods

### Data sources

The publicly available dashboard of FAERS was utilized to obtain data on reported adverse events from the first quarter of 2012 through the first quarter of 2023. ICIs included in the analysis were those available commercially in the United States at the time of the data pull (atezolizumab, avelumab, cemiplimab, dostarlimab, durvalumab, ipilimumab, nivolumab, nivolumab/relatlimab, pembrolizumab, and durvalumab/tremelimumab). Reaction terms utilized to select cases of ICI related myocarditis included myocarditis, autoimmune myocarditis, and immune mediated myocarditis. Following the acquisition of the dataset, cases were reviewed for duplications utilizing characteristics included in each individual report (reported age, weight, gender, event date, and cancer diagnosis). Identified cases were excluded if they were clearly duplications (one record of the duplicative information was retained). Additionally, cases were excluded if they did not include an age.

Data extracted from identified cases included: ICI drug, age, sex, year of report, region (North America, South America, Europe, Australia/New Zealand, Asia, Africa, not specified), and reported outcome (hospitalized, life threatening, death, other). All cases were reviewed and coded based on reported use of concomitant targeted therapy, concomitant chemotherapy, presence of additional reported non-cardiac immune related adverse events, and cancer diagnosis (breast, gastrointestinal, genitourinary, gynecologic, head/neck, hematologic, neurologic, other, skin, thoracic, or unknown).

### Statistical analysis

Reporting odds ratios (ROR) were calculated for each immune checkpoint inhibitor utilizing the 2x2 table in **Table 1** (5):

**Table 1:** 2x2 table for calculating ROR.

	Event of interest (myocarditis)	All other events
Immune checkpoint inhibitor of interest	A	C
All other products	B	D

ROR = (A*D)/(B*C)

Lower/upper bounds of 95% confidence interval (CI) of the ROR:

Lower bound = *e^Ln^* ^(ROR) - 1.96 [sqrt (1/a + 1/b +1/c + 1/d)]^
Upper bound = *e^Ln^* ^(ROR) + 1.96 [sqrt (1/a + 1/b +1/c + 1/d)]^

Nivolumab and ipilimumab cases were combined for calculation of ROR given the common utilization of these agents in combination and lack of individual review of all non-case (non-myocarditis) reports to determine which were reports of combination vs. monotherapy. The threshold for identification of a signal based on the calculated ROR was defined as a ROR greater than or equal to two and the lower bound of the 95% CI greater than one.

Descriptive statistics were utilized to describe group characteristics. A statistical analysis was completed to compare report characteristics based on outcome (hospitalized vs life threatening vs death vs other). Statistical tests employed included chi-squared test for nominal data and Mann-Whitney U test for non-parametric continuous data. Bonferroni correction was utilized in instances of multiple comparisons. Additional analysis were completed comparing those with a reported outcome of death and those with any other reported outcome, comparing characteristics and outcomes based on ICI therapeutic target, and comparing the use of combination or single ICI therapy. To assess the impact of time and experience on the outcomes of ICI related myocarditis, an analysis of event outcomes was completed comparing 2018 and 2022 (the last full year included in the dataset). Statistical comparisons were completed with IBM SPSS Statistics version 29.0.0.0.

## Results

**Figure 1:**
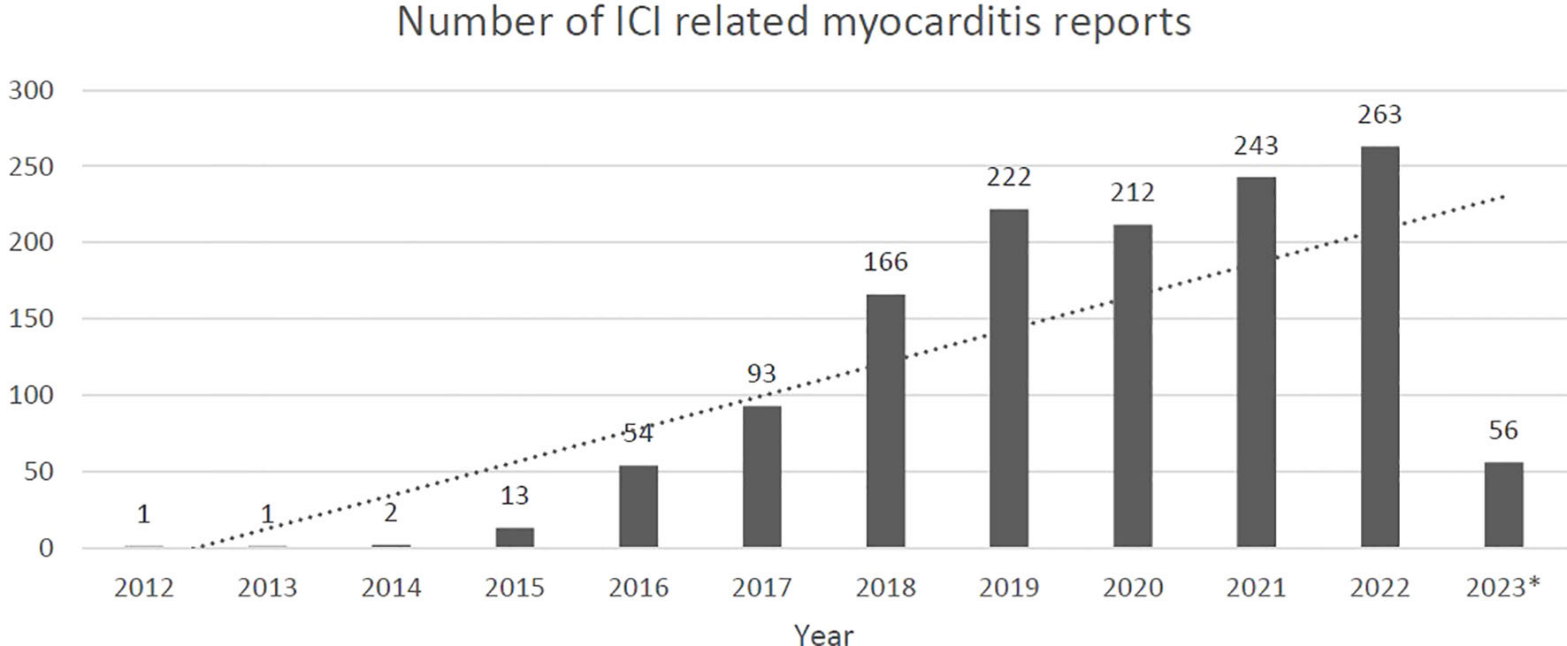
Reports of ICI related myocarditis by year *2023 data through the end of the first quarter 2023.

Between 2012 and the end of the first quarter of 2023, 20.7 million reports were identified in the FAERS system with 171,132 being reportedly related to the included ICI agents. Myocarditis with any agent accounted for 6,695 reports and when reports were selected based on relation to ICI use, 2,082 cases were identified. Upon exclusion of duplicate cases and those that did not include an age, 1,326 cases remained for analysis. In all, the included cases had a median age of 69 years (70% were over 65 years of age) and were majority male (62%). Reports most frequently included pembrolizumab (34%), nivolumab (28%), or nivolumab/ipilimumab combination (21%) as the offending ICI. Thoracic (29%), skin (24%), and genitourinary (20%) were the most frequently treated malignancies among the selected cases. Concomitant targeted therapy was reported to be utilized in 15% of cases and chemotherapy was reported in 12% of cases. For a complete listing of group characteristics, see **Table 2**. Reports of additional non-cardiac immune related adverse effects were present in 56% of the cases, the most commonly reported being myositis (n=346, 26%), hepatitis (n=215, 16%), and myasthenia gravis (n=179, 13%) (**Table 3**). As expected, given the continual uptake of ICIs for the treatment of multiple malignancies, the number of included ICI related myocarditis cases reported per year grew from one in 2012 to 263 in 2022 (the last full year included in the dataset) (**Figure 1**).

**Table 2:** Characteristics of included cases.

ICI product, n (%)		Cancer Diagnosis, n (%)	
Atezolizumab	102 (7.7)	Breast	23 (1.7)
Avelumab	19 (1.4)	Gastrointestinal	125 (9.4)
Cemiplimab	7 (0.5)	Genitourinary	260 (19.6)
Dostarlimab	2 (0.2)	Gynecologic	55 (4.1)
Durvalumab	36 (2.7)	Head/Neck	31 (2.3)
Durvalumab/Tremelimumab	15 (1.1)	Hematologic	12 (0.9)
Ipilimumab	26 (2.0)	Neurologic	7 (0.5)
Nivolumab	376 (28.4)	Other	19 (1.4)
Nivolumab/Ipilimumab	278 (21.0)	Skin	323 (24.4)
Nivolumab/Relatlimab	13 (1.0)	Thoracic	388 (29.3)
Pembrolizumab	452 (34.1)	Unknown	83 (6.3)
Sex, n (%)		Age, median (IQR)	69 (13)
Female	493 (37)	Weight, median (IQR)	70 (24)
Male	821 (62)	Concomitant Targeted Therapy, n (%)	205 (15)
Not specified	12 (1)	Concomitant Chemotherapy, n (%)	161 (12)
Year, n (%)		Region, n (%)	
2012	1 (0.2)	North America	376 (28)
2013	1 (0.2)	South America	11 (1)
2014	2 (0.2)	Europe	466 (35)
2015	13 (1.0)	Australia/New Zealand	48 (4)
2016	54 (4.1)	Asia	352 (27)
2017	93 (7.0)	Africa	1 (0)
2018	166 (12.5)	Not specified	72 (5)
2019	222 (16.7)		
2020	212 (16.0)		
2021	243 (18.3)		
2022	263 (19.8)		
2023	56 (4.2)		

**Table 3:** Concurrent non-cardiac immune related adverse event included in report.

	N (%)
Any concurrent non-cardiac IRAE	736 (56)
Top 10 reported concurrent non-cardiac immune related adverse events*	
Myositis	346 (26)
Hepatitis	215 (16)
Myasthenia Gravis	179 (13)
Thyroiditis	85 (6)
Nephritis	76 (6)
Pneumonitis	74 (6)
Rash	67 (5)
Colitis	65 (5)
Hypophysitis	37 (3)
Diabetes (type 1)	22 (2)

*More than one IRAE may have been reported per case.

**Figure 2:**
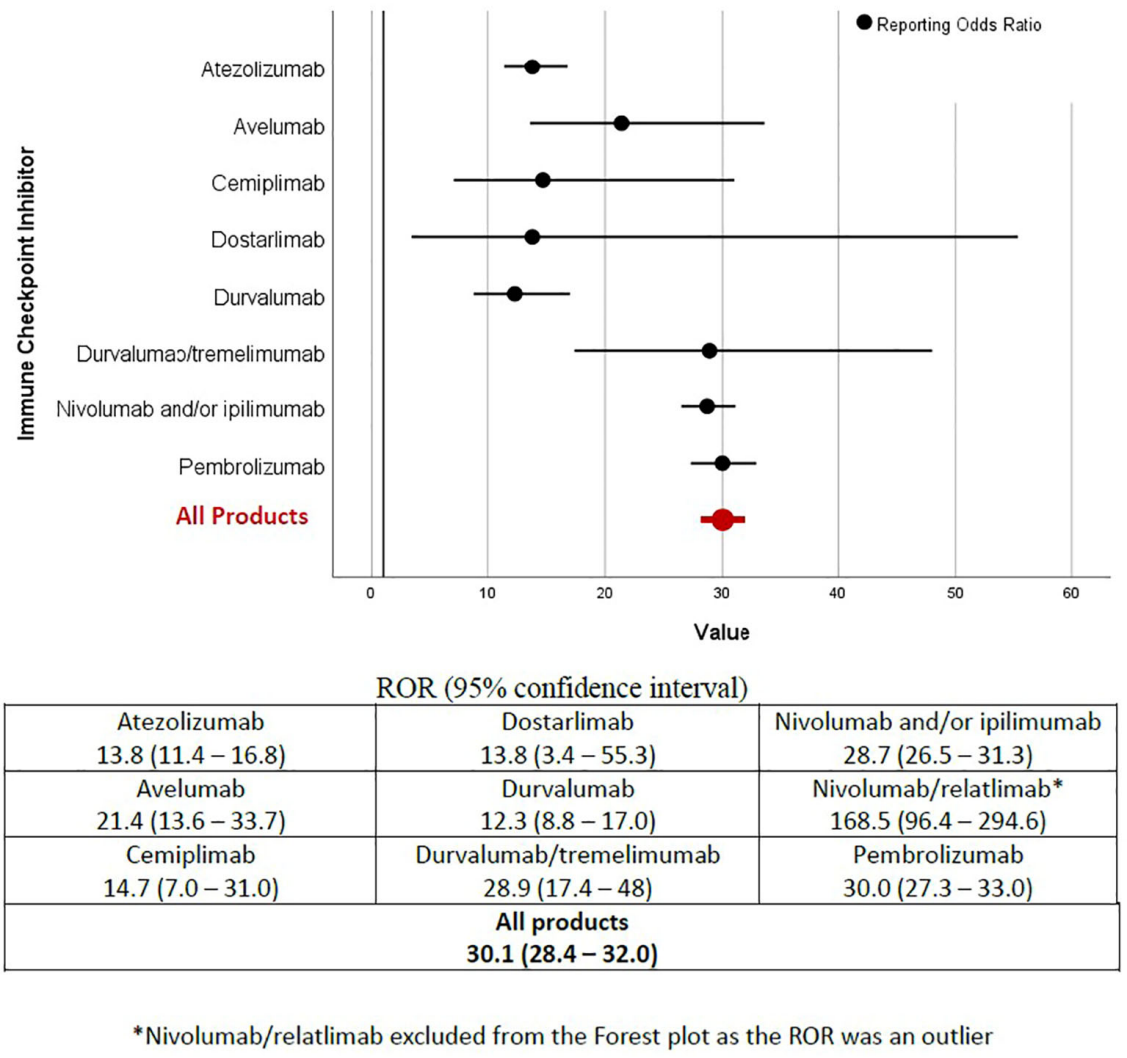
Reporting Odds Ratio. ROR (95% confidence interval).

All nine ICIs included were associated with myocarditis reports and the overall ROR for all products combined was 30.1 [95% confidence interval: 28.4-32.0] (**Figure 2**). When excluding the outlier nivolumab/relatlimab (ROR 168.5, 95% CI: 96.4-294.6), likely due to the limited number of reported events, the agents with the strongest associations were with pembrolizumab (ROR 30.0, 95% CI: 27.3 – 33.0), durvalumab/tremelimumab combination (ROR 28.9, 95% CI: 17.4-48), and nivolumab and/or ipilimumab (ROR 28.7, 95% CI: 26.5 – 31.3). Interestingly, the durvalumab/tremelimumab ROR was greater than twice the ROR of durvalumab monotherapy (28.9 vs 12.3, respectively) and their 95% confidence intervals did not overlap (**Figure 2**). In fact, durvalumab monotherapy had the lowest ROR, followed by dostarlimab, cemiplimab, and atezolizumab. Based on the calculated RORs, ICIs appear to fall into two groups: those with ROR < 15 (durvalumab, dostarlimab, cemiplimab, atezolizumab) and those with ROR > 25 (durvalumab/tremelimumab, nivolumab and/or ipilimumab, pembrolizumab). Avelumab falls between the two groups with an ROR of 21.4.

The most common reported outcome of ICI related myocarditis in the included cases was death (37%) followed by hospitalization (31%), life-threatening (21%), and other (11%). In comparing the characteristics of cases with a reported outcome of death to those not reporting death as the outcome, death occurred less frequently in cases receiving concomitant targeted therapy (10% vs. 19%, p<0.001) and those with a fatal outcome were older (median age: 70 years vs. 69 years, p=0.02). Significant differences between groups were present for reporting year and reporting region and after pairwise analysis with Bonferroni correction, statistical significance remained for the 2015 cohort and those reported in North America, South America, and Australia/New Zealand. To determine if improvements in outcomes emerged with increasing experience with ICIs over time, a comparison of outcomes in 2018 and 2022 was completed, and fatality rates were significantly lower in 2022 (fatality rate 33% vs 45%, p=0.017). No differences were observed between groups in sex, concomitant chemotherapy, concurrent non-cardiac IRAE, and ICI product. See **Table 4** for the complete analysis of case characteristics based on outcome.

**Table 4:** Comparison of characteristics between cases with reported outcome of death and other outcome.

Characteristic	Other Outcome (n=829)	Death (n=497)	P value
Male, n (%)	495 (60)	326 (66)	0.087
Age, median (IQR)	69 (14)	70 (14)	0.02
Concomitant targeted therapy, n (%)	156 (19)	49 (10)	<0.001
Concomitant chemotherapy, n (%)	100 (12)	61 (12)	0.909
Concurrent non-cardiac IRAE, n (%)	449 (54)	287 (58)	0.204
Report year, n (%)			0.045
2012	1 (100)	0 (0)	
2013	1 (100)	0 (0)	
2014	1 (50)	1 (50)	
2015	4 (31)	9 (69)*	
2016	29 (54)	25 (46)	
2017	52 (56)	41 (44)	
2018	92 (55)	74 (45)	
2019	144 (65)	78 (35)	
2020	128 (60)	84 (40)	
2021	164 (67)	79 (33)	
2022	176 (67)	87 (33)	
2023	37 (66)	19 (34)	
ICI product, n (%)			0.133
Atezolizumab	77 (75)	25 (25)	
Avelumab	14 (74)	5 (26)	
Cemiplimab	4 (57)	3 (43)	
Dostarlimab	2 (100)	0 (0)	
Durvalumab	24 (67)	12 (33)	
Durvalumab/tremelimumab	8 (53)	7 (47)	
Ipilimumab	15 (58)	11 (42)	
Nivolumab	220 (59)	156 (41)	
Nivolumab/ipilimumab	166 (60)	112 (40)	
Nivolumab/relatlimab	9 (69)	4 (31)	
Pembrolizumab	290 (64)	162 (36)	
Region, n (%)			<0.001
North America	259 (69)	117 (31)*	
South America	6 (55)	5 (45)	
Europe	263 (56)	203 (44)*	
Australia/New Zealand	39 (81)	9 (19)*	
Asia	209 (59)	143 (41)	
Africa	1 (100)	0 (0)	
Not specified	52 (72)	20 (28)	

*After Bonferroni correction, a statistically significant difference remained for these variables compared to other variables in this analysis after pairwise analysis.

In the analysis of characteristics based on therapeutic target (**Table 5**), concomitant therapy was more common with PD-L1 therapies and concurrent non-cardiac IRAEs were more commonly reported with PD-1 therapies. There was a significant difference in the reported outcome with death being a more common outcome among the PD-1 therapies compared to the PD-L1 therapies after Bonferroni correction (38% vs. 27%). Assessing the reports for combination ICI therapy compared to single agent therapy (**Table 5**), cases involving combination therapy were significantly younger and less likely to receive concomitant therapy. There was no difference in the reported outcome of death between cases of single agent therapy or combination therapy (37% vs. 40%, p=0.263).

**Table 5:** Analysis of report characteristics and outcome by therapeutic target and use of combination or single ICI therapy.

Therapeutic Target (excluding patients receiving combination therapy)				
Characteristic	PD-L1 (n=157)	PD-1 (n=837)	CTLA-4 (n=26)	P value
Male, n (%)	96 (61)	515 (62)	17 (65)	0.970
Age, median (IQR)	69 (15)	70 (14)	71 (15)	0.332
Concomitant targeted therapy, n (%)	76 (48)^a^	111 (13)^b^	1 (4)^b^	<0.001
Concomitant chemotherapy, n (%)	36 (23)^a^	97 (12)^b^	1 (4)^a,b^	<0.001
Concurrent non-cardiac IRAE, n (%)	71 (45)^a^	466 (56)^b^	16 (62)^a,b^	0.041
Reported outcome: Death	42 (27)^a^	321 (38)^b^	11 (42)^a,b^	<0.001
Single ICI based regimen vs. Combination ICI based regimen				
	Single Agent ICI Regimen (n=1020)	Combination ICI Regimen (n=360)		P value
Male, n (%)	628 (62)	193 (63)		0.872
Age, median (IQR)	70 (14)	68 (13)		0.034
Concomitant targeted therapy, n (%)	188 (18)	17 (6)		<0.001
Concomitant chemotherapy, n (%)	134 (13)	27 (9)		0.043
Concurrent non-cardiac IRAE, n (%)	553 (54)	183 (51)		0.084
Reported outcome: Death	374 (37)	123 (40)		0.263

^a^, ^b^, After Bonferroni correction, subscript letters denote where the statistical significant difference was identified after pairwise analysis (if the subscript letter is the same, proportions do not differ significantly from each other).

## Discussion

To our knowledge, this analysis includes the largest number of ICI related myocarditis cases to date (**Table 6**) (5–7). Though all included ICIs were associated with myocarditis, interesting trends are appearing, including in the reported fatality rates. Likewise, the number of reported myocarditis cases have increased each year, which must be interpreted with caution given the lack of a denominator and the continuous expansion in utilization (i.e., the increasing number of reports likely represents an increase in utilization of ICIs instead of an increase in risk for myocarditis). Remarkably, the 1,326 identified ICI related myocarditis reports account for almost 20% of all myocarditis reports submitted to the database during the time included. Compared to prior studies utilizing the FAERS database, our analysis includes more recent data reflecting current trends in ICI utilization (5–7). For example, our analysis is the only to include more recently approved agents such as relatlimab (utilized in combination with nivolumab) and tremelimumab (utilized in combination with durvalumab). Furthermore, compared to the 2004 – 2018 analysis of FAERS, our analysis included greater than 5 times the number of pembrolizumab, (452 vs. 69, respectively), atezolizumab (102 vs 18, respectively), and avelumab (19 vs 4, respectively) cases and twelve times the number of durvalumab cases (36 vs. 3, respectively), consistent with trends in ICI utilization. Similarly, the most utilized ICIs in the 2004 – 2018 analysis were nivolumab and/or ipilimumab, whereas the most utilized agent in our analysis was pembrolizumab.

**Table 6:** Comparison of prior FAERS database analyses of ICI myocarditis reports.

Report	Years included	Number of reports included	Number of ICI reports included	Number of myocarditis reports included	Number of ICI myocarditis reports included
Current study	2012 – Q3 2023	20.7 million	171,132	6,695	1,326
Fan Q, et al (5)	2004 – 2018	9.35 million	43,147	4,007	315
Makunts T, et al. (6)	2004 – Q2 2020	14.2 million	61,961		
Ma R, et al. (7)	2014 - 2019		5,786*	610*	

*Only represents fatal cases. Cells grayed out include data not presented or quantifiable in reference.

The majority of ICI related myocarditis cases in our analysis were reported in males (62%). This is similar to the analysis of FAERS data from 2004 – 2018 which included 58% males (5). The preponderance of ICI related adverse effects among males is further supported by an analysis of all immune-related adverse events reported to FAERS between 2004 and 2020 (8). In that analysis, almost twice the number of reports were submitted for males compared to females (19,245 males vs. 11,097 females). While not limited to myocarditis, when cardiovascular toxicity was analyzed, there was a signal for toxicity in males compared to females after 1:1 propensity score matching of report characteristics (proportional reporting ratio 2.25, 95% CI: 2.07-2.45). Despite this similarity with previously published data, the age of cases included in our report trended older than in the prior study of cases from 2004-2018 (≥ 65 years: 70% vs 51%, respectively) (5). This is to be expected due to changes in utilization trends and inclusion of cases through the first quarter of 2023 in our analysis. Since 2018, ICI indications have expanded to include the vast majority of lung cancer cases, triple negative breast cancer cases, and as first-line treatment for multiple malignancies (kidney, head/neck, hepatocellular, esophagogastric, gynecologic and biliary cancers) (1). Prior to this expansion, ICIs had a limited number of first-line indications and their use was well established in melanoma, a malignancy with a younger age distribution. In addition to the substantial increase in indications since 2018, multiple newer agents have come to the market during this time (dostarlimab, tremelimumab, and relatlimab), further expanding their use. In comparing the reported indications between the analysis of cases through 2018 and our analysis, thoracic malignancies were the most common in both (37% vs 29%, respectively). Our analysis had a lower proportion of patients with skin tumors (30% vs 24%, respectively) and a higher proportion with genitourinary tumors (12% vs 20%, respectively), gastrointestinal tumors (5% vs 9%, respectively), and gynecologic tumors (0.3% vs 4.1%). These alterations in utilization patterns over time could account for differences in characteristics (such as age) between the two overlapping cohorts.

Regardless of any differences observed, all ICIs included in the varying analyses, including our analysis, have demonstrated an association between ICIs and myocarditis (5–7). The ROR for the class as a whole in our analysis was 30.1 and was similar to pembrolizumab and nivolumab and/or ipilimumab RORs, likely due to the fact that those ICIs contributed the majority of cases to the dataset. Nivolumab/relatlimab was an outlier with a ROR of 168.5 (5.8 times higher than that observed with nivolumab and/or ipilimumab). This may be explained by the limited number of cases; however, it should be noted that durvalumab/tremelimumab combination ROR was twice that of durvalumab monotherapy. Again, this comparison is limited by the number of cases in both groups, but dual ICI therapy may be associated with higher RORs for myocarditis. This is further reinforced by the fact that durvalumab and durvalumab/tremelimumab ROR 95% CIs did not overlap. This phenomenon was also present in the prior analysis through 2018 where ipilimumab plus pembrolizumab and ipilimumab plus nivolumab had higher RORs than ipilimumab, nivolumab, or pembrolizumab alone (5).

Our analysis is limited in that it combined nivolumab and ipilimumab for analysis. Further limiting the analysis is the fact that the above cited odds ratios are reporting odds ratios and dependent on reporting to the FAERS database; therefore, it can be difficult applying this to clinical practice. Nevertheless, these data confirm the ongoing association between ICIs and myocarditis. In an analysis of Integrated Summaries of Safety (ISS) which includes information from clinical trials, the odds ratio for myocarditis was non-significant at 3.6 (95% CI 0.5-27.1) (6). The lack of significance was likely due to the lack of cases of myocarditis reported (16 cases among 20,062 ICI treated subjects; 1 case among 4,505 control group subjects), highlighting the importance of databases such as FAERS and analyses such as this to identify safety signals despite the inherent limitations in utilizing reporting databases.

As discussed above, dual ICI therapy has been established as a likely risk factor for ICI myocarditis. An additional risk factor is the presence of a concurrent iRAE (2, 4). Our analysis identified a concurrent iRAE reported with 56% of the cases. This is similar to data from 101 cases reported to the World Health Organization database (VigiBase) which identified a concurrent iRAE reported in 42% of cases (9). They also identified myositis in 25% and myasthenia gravis in 11%, similar to the rates identified in our analysis (26% and 13%, respectively).

Outcomes of reported cases appear to be improving as we gain more experience with ICIs and the management of their toxicities. The fatality rate for reported myocarditis cases in our analysis was 37%, down from 55% in the analysis from 2018 (5). This reduction in fatality rate is further supported by our analysis of outcome in 2018 and 2022 and the significant reduction in fatality over time. Interestingly, the fatality rate was significantly lower in those receiving concomitant targeted therapy. Despite a potential increase in risk for ICI related adverse effects overall when utilized in combination with targeted therapy, as demonstrated in an analysis of adverse event cases reported to FAERS from 2012 to 2021 for those receiving a combination of bevacizumab and ICI, our data suggests the fatality rate may be lower among those receiving combinations with targeted therapy (10). Another comparison of adverse events reported to FAERS or Vigibase between nivolumab/ipilimumab and nivolumab/cabozantinib demonstrated a disproportion in myocarditis between the groups with the nivolumab/ipilimumab demonstrating a potentially higher risk (11). The potential decrease in fatality with concomitant targeted therapy may be due to the fact that combination therapy is a more recent development and may be a surrogate for when the treatment was administered (there may have be improvements in outcomes more recently as demonstrated by the fatality rate over time). However, this does not address the decreased risk for myocarditis suggested by the analysis of nivolumab/cabozantinib. Another trend that appears to be emerging is the improved outcomes in North America, Europe, and Australia compared to Asia and South America. This data must be interpreted cautiously as there were relatively few cases in some groups (i.e., South America N=11, Africa, N=1).

When assessing based on therapeutic target, interesting trends emerged in that the reported outcome was more likely to be death in reports of agents targeting PD-1. Likewise, reports of PD-1 were less likely to be receiving concomitant targeted therapy which was a trend among the entire population in that those reporting a concomitant targeted therapy were less likely to have the outcome of death. It is unknown whether this increase in reports of death were related to the therapeutic target being PD-1 or if there was an impact of the decreased frequency in use of concomitant targeted therapy. Despite an increase in the ROR associated with combination ICI therapy, there was no increase in reports of death for combination therapy compared to single agent therapy.

In a meta-analysis of ICI-related cardiotoxicity, 40% of those experienced cardiovascular iRAEs had cardiovascular risk factors with the most common being myocardial infarction, peripheral coronary artery disease, and hypertension (12). Furthermore, a multivariable analysis of major cardiovascular adverse events in patients treated with ICIs demonstrated that a history of heart failure and valvular heart disease were risk factors for MACE (13). Based on the risk for poor outcomes and the association with preexisting cardiovascular disease among patients with ICI-induced myocarditis, the European Society of Cardiology (ESC) developed recommendations for monitoring patients receiving ICI therapy (14). Prior to therapy initiation, the ESC guidelines recommend a baseline electrocardiogram (ECG) and troponin level. During therapy, it is recommended that periodic ECG, troponin, and natriuretic peptide levels be monitored. High risk patients should also have a baseline echocardiogram completed. If ECG abnormalities, elevations in cardiac biomarkers, or cardiac symptoms are present, an echocardiogram should be completed to investigate for myocarditis. Given the concurrence of myocarditis with myositis and myasthenia gravis, patients experiencing these other iRAE should also be monitored more closely for the development of cardiac symptoms with a low threshold for obtaining an echocardiogram to identify myocarditis in symptomatic patients. In an effort to provide continued improvement in outcomes and to advance the management of ICI related myocarditis, a phase 3 trial of abatacept compared to placebo in conjunction with high dose steroids in the management of myocarditis secondary to ICI is underway (ATRIUM trial, NCT05335928) (15). National Comprehensive Cancer Network (NCCN) guidelines for the treatment of ICI related myocarditis recommend discontinuation of the ICI therapy and initiation of high doses of methylprednisolone (1g/d for 3-5 days followed by a slow taper) (16). For patients not improving on steroids after 24 to 48 hours, NCCN guidelines recommend further immunosuppression, yet they do not give preference to any one agent. Options include abatacept, alemtuzumab, antithymocyte globulin, infliximab, intravenous immunoglobulin, methotrexate, and mycophenolate. The ATRIUM trial described above will provide the first randomized trial assessing an intervention for the treatment of ICI related myocarditis.

## Conclusion

Though rare, ICIs are associated with a risk for myocarditis which can be fatal. Over time, it appears that the fatality rate is declining as awareness increases and advances are made in the understanding and treatment of this relatively rare immune related adverse effect. Through adherence to recommended monitoring and a low threshold to investigate symptomatic patients, particularly those with concomitant myositis or myasthenia gravis, outcomes may continue to improve. The results of the first randomized clinical trial in ICI related myocarditis is awaited with the potential to further improve outcomes in this growing population of patients.

## Data Availability

Publicly available datasets were analyzed in this study. This data can be found here: https://fis.fda.gov/sense/app/95239e26-e0be-42d9-a960-9a5f7f1c25ee/sheet/7a47a261-d58b-4203-a8aa-6d3021737452/state/analysisFDA Adverse Event Reporting System (FAERS) Public Dashboard.
